# A Comprehensive Review of Pediatric Necrotizing Pneumonia

**DOI:** 10.3390/children12091248

**Published:** 2025-09-17

**Authors:** Manette Ness-Cochinwala, Balagangadhar R. Totapally

**Affiliations:** 1 Division of Critical Care Medicine, Nicklaus Children’s Hospital, Miami, FL 33155, USA; manette.ness-cochinwala@nicklaushealth.org; 2 Department of Pediatrics, Herbert Wertheim College of Medicine, Florida International University, Miami, FL 33199, USA

**Keywords:** pneumonia, lung necrosis, empyema, complicated pneumonia

## Abstract

**Highlights:**

**What are the main findings?**

- The epidemiology, microbiology, and current management of necrotizing pneumonia in children are reviewed.
- The prevalence of necrotizing pneumonia in children is increasing.

**What is the implication of the main finding?**

- Awareness of the rising prevalence and shifting microbiological patterns can facilitate early recognition and guide appropriate antimicrobial selection.
- Thoracostomy with fibrinolytics is more often preferred compared to operative therapies for managing empyema associated with necrotizing pneumonia in children.

**Abstract:**

Necrotizing pneumonia is a serious complication of pediatric pneumonia, characterized by liquefaction and cavitation of the lung parenchyma. *Streptococcus pneumoniae* and *Staphylococcus aureus* are the most implicated organisms. *Mycoplasma pneumoniae* has been an increasingly recognized pathogen, especially is Asian and *Pseudomonas aeruginosa* is mainly noted in a higher percentage of patients with complex chronic conditions. Clinical presentation typically includes fever, respiratory distress, and failure to respond to standard antibiotic therapy. These patients are more likely to have pleural involvement in the form of effusion or empyema and a higher need for respiratory support. Diagnosis is typically through a combination of chest radiographs, lung ultrasound, and chest computed tomography. Management is primarily via prolonged intravenous antibiotics that cover the above organisms, though pleural drainage with fibrinolytics is often required. Surgical intervention is often reserved for refractory cases that fail initial fibrinolytic therapy. Prognosis is usually favorable in the short and long term, though early recognition and appropriate management are imperative to reduce the duration of illness and morbidity.

## 1. Introduction

Although there has been an overall decrease in the number of lower respiratory illnesses globally over the last few decades, there is a substantial burden, especially in low and middle-income countries [1]. Pneumonia can be classified as community-acquired pneumonia (CAP) or hospital-acquired pneumonia (HAP). Ventilator-associated pneumonia (VAP) is mostly a form of HAP that occurs at least 48 h after intubation. Aspiration pneumonia can be CAP or HAP. Due to a lack of clear guidelines for diagnosis, the true incidence of aspiration pneumonia among children with CAP and HAP is not known [2]. The classification of various types of pneumonias is presented in Table 1.

Complicated pneumonia is another term used in the literature, often described for any pneumonia associated with parapneumonic effusion (PPE), empyema, necrotizing pneumonia (NP), or lung abscess [9]. Community or hospital-acquired pneumonia can result in necrosis of the lung parenchyma, leading to necrotizing pneumonia. Lung abscess, NP, and pulmonary gangrene (lung necrosis) are a part of a spectrum of disease with varying degrees of necrosis and varied presentations (Table 2) [10,11].

NP is often discovered on chest radiograph, chest ultrasound (U.S.), or computerized tomography (CT) performed in children with pneumonia who are ill appearing or with prolonged fever [12]. The clinical outcomes of NP range from mild to life-threatening, depending on the lung necrosis severity and associated complications such as empyema, pneumatoceles, lung abscess, and bronchopleural fistula [12,13]. Though these children are often acutely ill, requiring prolonged hospitalization with a higher rate of invasive procedures, in healthy children, NP usually resolves with appropriate antimicrobial treatment and is associated with a good long-term prognosis [14,15]. The aim of this article is to provide a comprehensive review of the clinical presentation, diagnosis, and management of necrotizing pneumonia.

## 2. Epidemiology

Necrotizing pneumonia is an uncommon but increasingly recognized complication of pediatric pneumonia, accounting for 3–7% of hospitalized cases of pediatric CAP and up to 40% of complicated pneumonias [14,15,16,17,18,19,20]. High prevalence of NP is noted in some series. In children hospitalized with CAP in Egypt, 54% were diagnosed with NP, and 64% of children with NP were associated with empyema [21]. In a study from three major hospitals in Jerusalem in the years 2001–2010, out of 144 children admitted with CAP, empyema and NP were present in 40% and 20%, respectively [22]. Recent database and multicenter studies have demonstrated a rising incidence, likely due to a combination of an actual rise in incidence as well as improved detection with advanced imaging modalities [16,23,24]. Incidence is higher in the winter and spring months [21]. Most patients with CAP, as well as complicated pneumonia, are admitted during the influenza season [9].

The median age at presentation is most commonly between 3 and 5 years, but can present at any age [14,17,21,25,26]. There is no gender predilection [27]. The majority affected are previously healthy; however, those with complex chronic conditions may experience more severe disease and higher mortality rates [14].

## 3. Pathophysiology

**Figure 1:**
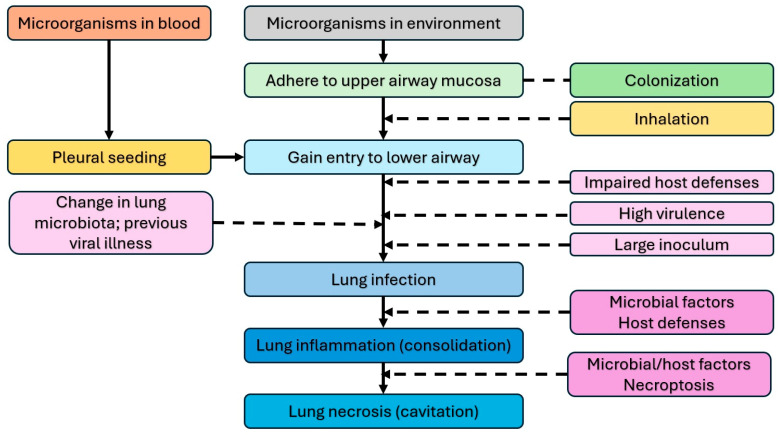
Pathogenesis of lung infection, inflammation, and necrotizing pneumonia [2].

Necrotizing pneumonia is an infection with severe inflammation of a region of alveoli or a lung lobe, leading to severe tissue damage and necrosis. Typically, it results from pulmonary infection, causing destruction and liquefaction of lung tissue, with loss of normal parenchymal architecture. The pathogenesis of NP involves several factors that lead to lung infection, including impaired host defense mechanisms (e.g., immunosuppression, loss of barrier function, etc.), high virulence of microorganism, large microbial inoculum, and change in normal lung microbiota (e.g., previous viral illness) [2]. This leads to pneumonia and consolidation. In some severe cases, liquefaction of consolidated areas occurs, leading to necrotizing pneumonia. Necroptosis is the primary mechanism for liquefaction and necrosis [28]. Figure 1 shows a schematic representation of the pathogenesis of lung infection and the development of NP. In rare circumstances, septic emboli (e.g., from endocarditis of the right heart, venous line infections, etc.) can seed and occlude pulmonary arteries, leading to necrotic areas in the lungs [5].

The mechanism of NP has not been fully elucidated, but it is thought to be a combination of vasculitis and thrombosis of intra-pulmonary vessels leading to ischemia, liquefaction, and necrosis of the affected lung [13]. The decreased blood supply leads to decreased antibiotic penetration of the affected tissue, which is why the infection may spread to the pleural space or not respond well to antibiotics initially [29].

### Necroptosis

Necroptosis is a regulated cell death that shares the morphological characteristics of apoptosis and necrosis. It presents as cellular swelling and plasma membrane rupture [28]. Various microbial products and host reactions to them lead to necroptosis in the lung, which further propagates inflammation [28]. Infection with some microorganisms is more likely to lead to liquefaction and necrosis (Table 3).

**Figure 2:**
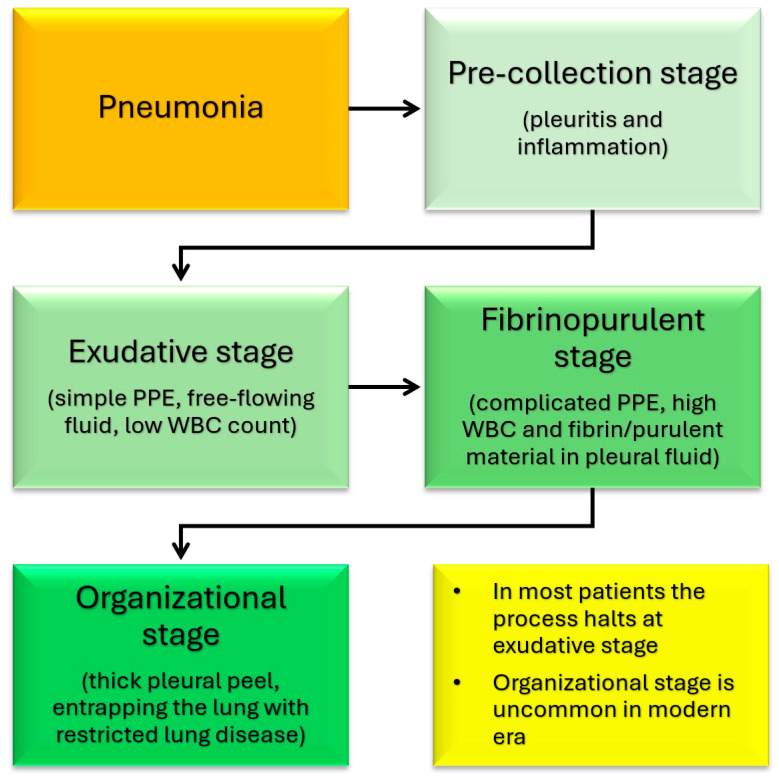
Various stages of development of pleural inflammation, parapneumonic effusion (PPE), and empyema associated with pneumonia.

This combination of factors leads to liquefication and cavitation of the lung parenchyma [12,13,35]. The cavitation can create single or multiple thin-walled cavities known as pneumatoceles, which are common in NP. Pneumatoceles are generally peripheral and in one lobe [36]. These small cavities can combine to form large cysts with air-fluid levels or rupture into the pleural space, leading to a bronchopleural fistula [13,37]. Pleural involvement is common with parapneumonic effusions, empyema, and bronchopleural fistulas occurring at a much higher rate than in non-necrotizing pneumonia [16,17,21,23]. The development of PPE and empyema is described to occur in four stages (Figure 2) [38]. The initial stage of pleurisy is followed by simple PPE, then complex PPE (empyema), and finally thick pleural plaques. In most patients, the process halts at the stage of simple PPE. In the modern era, pleural inflammation rarely leads to the stage of thick pleural plaques [38].

In 1985, Light proposed criteria for diagnosing an exudative parapneumoic effusion, where meeting one of the criteria is indicative of exudative fluid (Table 4) [39,40]. He later revised his proposal to seven stages for escalating therapeutic options for effusions related to pneumonia [41]. Pleural fluid examination has been shown to help define the severity of PPE [42,43,44]. A two-day course of antibiotics before pleural fluid examination has been shown to decrease the yield of culture but not affect biochemistry [45]. The American College of Chest Physicians has published staging of pleural infection and drainage recommendations (Table 5) [46]. However, the evidence for pleural fluid classification and correlation with treatment benefits is weak [38].

## 4. Clinical Course

The clinical outcomes of NP range from mild to life-threatening, depending on the lung necrosis severity and associated complications [15]. Presentation is typically persistent fever, cough, and respiratory distress with a failure to improve after 2–3 days of antibiotics [13,23,47]. Pleural involvement is almost universal, with up to 90% of patients having an effusion or empyema [16,17,21,25,27]. Fever can persist in these patients for a mean of 9–16 days after admission [22,48].

Overall, they present with typical symptoms of pneumonia, though they are much sicker and fail to respond adequately to initial antibiotics [23]. However, some children can present with fulminant pneumonia that progresses to respiratory failure and shock [15]. Systemic complications like sepsis, septic shock, hemolytic uremic syndrome, and, less commonly, multiorgan dysfunction syndrome can also occur, especially in younger children or those with co-morbidities [13,14,21].

Hospitalization tends to be prolonged, especially in comparison to non-necrotizing pneumonia. The median length of stay ranges from 12 to 26 days, with a significant percentage requiring pediatric intensive care unit admission [16,17,21,25,26]. Invasive interventions are also common, with 40–90% of children requiring pleural drainage and a minority requiring a surgical procedure such as decortication or resection [14,16,17,21,25]. This level of pleural drainage is consistent with the high level of pleural involvement in these patients. Mechanical ventilation is required in up to 28% of cases, and complications like pneumothorax and bronchopleural fistula are not infrequent [14,21].

Mortality rates are generally low (0–5%) but are higher in infants and in those with underlying chronic conditions [14,21,25]. In one study, about a third of patients with NP, lung abscess, or giant lung cyst were observed, and all were treated conservatively with good outcomes [37]. Despite severity and prolonged course, long-term outcomes are favorable for most children [15,16].

## 5. Etiology

Bacterial pathogens dominate the microbiologic etiology of pediatric NP. The causative pathogen is often not identified, likely due to prior antibiotic use. In fact, less than 10% of blood cultures are positive [15]. *Streptococcus pneumoniae*, *Staphylococcus aureus*, and other bacteria, fungi, and viruses can cause NP [23]. Mycoplasma has become a more common cause of pneumonia and NP in recent years, especially in China. Non-microbial processes, such as aspiration of gastric contents, medications, and collagen vascular diseases, can also cause NP [23]. Several other less common causes include Yersinia, Legionella, Hanta virus, tuberculosis, and others [49]. Some of the organisms causing NP are listed in Table 3.

*Streptococcus pneumoniae* is the most frequently identified causative agent in culture-positive cases in both retrospective and prospective studies [12,15]. Often found via pleural fluid or lung aspirate. It remains the leading pathogen even in the post-pneumococcal conjugate vaccine era. Pneumococcal necrotizing pneumonia is associated with rapid progression to necrosis with a high incidence of pleural complications such as empyema and parapneumonic effusion [14,16,17,23,25,50].

The introduction of pneumococcal vaccination has changed the prevalence of serotypes. In a study from a children’s hospital in Utah, USA, during the period 1997–2000, the incidence of NP increased in culture-proven pneumococcal pneumonia. Serotype 3 was the predominant pneumococcal type in children with NP [51]. This serotype was not included in the 7-valent conjugate vaccine, which was available during this study period. Serotype 3 infection was common in children with NP in a report from Spain [52]. The introduction of the 13-valent pneumococcal conjugate vaccine (PCV13) has decreased the relative frequency of invasive pneumococcal infection with serotype 3 [53]. After the introduction of PCV13 in France, the prevalence of NP in a hospital has not changed; however, the relative frequency of isolation of *Streptococcus pneumoniae* has decreased [54]. In the U.S., there is a decrease in the prevalence of empyema in children after the introduction of PCV13, especially in children under 2 years of age [55].

*Staphylococcus aureus* is the second most common pathogen, with both methicillin-sensitive (MSSA) and methicillin-resistant (MRSA) strains implicated [56]. Staphylococcal NP is a severe disease with a high mortality [57]. The emergence of community-acquired MRSA, particularly strains with the Panton-Valentine leucocidin (PVL) gene, has been associated with severe, rapidly progressive NP with increased morbidity, including higher rates of respiratory failure, bronchopleural fistula, and need for intensive care. PVL-positive *Staphylococcus aureus* (PVL-SA) commonly causes recurrent skin abscesses and furunculosis. However, it can also lead to severe, life-threatening infections, including NP [58]. PVL-positive *S. aureus* is associated with extensive parenchymal destruction and systemic toxicity [14,21,23,59,60,61]. PVL creates pores in the mitochondrial and cell membranes of neutrophils and macrophages, which induces cell apoptosis and breakdown. This causes a release of inflammatory mediators, which is thought to lead to the increased clinical severity [62]. PVL-SA, coinfection with influenza, and leukopenia are associated with poor outcomes in community-acquired pneumonia [63].

A study using the MRSA surveillance database of Beijing Children’s Hospital reported an increase in the incidence of pediatric invasive MRSA infections among Chinese children between 2006 and 2011, with younger children more commonly affected and presenting with more severe pneumonia or empyema [64]. The effect of Methicillin resistance on outcomes from Staphylococcal infections is variable. A study from France found that methicillin resistance was not associated with increased severity of staphylococcal NP [57]. However, in a single-center study from a PICU, community-acquired invasive MRSA infections were associated with high mortality, extended hospital stays, and 55% had NP [65].

*Mycoplasma pneumoniae* is an increasingly recognized cause of pediatric NP, particularly in East Asian cohorts [66]. In some areas of China, *Mycoplasma pneumoniae* has become the most common agent for NP in children [27]. NP caused by *Mycoplasma pneumoniae* is associated with a prolonged indolent clinical course, severe laboratory and radiologic features [67]. Although it tends to have a more indolent course compared to more typical bacterial NP, it can still result in significant parenchymal necrosis. Distinguishing features include a high incidence of unilateral, unilobar involvement and a lower frequency of pleural effusion [27,68,69]. Consolidation or atelectatic pattern on chest radiograph in patients with *Mycoplasma pneumoniae* infection is associated with NP [66,70]. Time to develop and detection of necrosis is longer with *Mycoplasma pneumoniae* [27,66].

The presence of Mycoplasma in pleural fluid in children with pneumonia from *Mycoplasma pneumoniae* with pleural effusion leads to a more serious clinical course [71]. *Mycoplasma pneumoniae* pneumonia in patients with medium to large pleural effusions or lung necrosis leads to a more severe course and takes a longer time for radiologic recovery [20]. Serum D-dimer levels may be helpful in identifying who will develop these pulmonary complications [20]. Elevated lactate dehydrogenase levels are observed in pneumonia caused by *Mycoplasma pneumoniae* and may aid in the early detection of necrotizing pneumonia due to this organism [72]. Macrolide resistance is associated with a more severe clinical course and increased complications and should be considered in cases of severe NP caused by *Mycoplasma pneumoniae* [73,74]. Outcomes of children with NP due to *Mycoplasma pneumoniae*, in general, are good, although they require prolonged hospital stays [66].

Group A Streptococcus (GAS) infection was the most commonly isolated organism in community-acquired pneumonia in Warsaw, Poland, in the post-COVID-19 era [75]. The duration of chest tube drainage was shorter with GAS compared to other causes of complicated pneumonia [75].

Other less common pathogens include *Fusobacterium nucleatum*, *S. pyogenes* (especially after influenza illness), β-hemolytic streptococci (Lancefield groups C and G) and in children with complex chronic conditions, *Pseudomonas aeruginosa* [14,76]. Similarly to other *Staphylococcal pneumonias*, preceding viral illness is a significant risk factor [12,14,50,60,77].

Viral infections: Respiratory syncytial virus (RSV) is a leading cause of acute lower respiratory infections (ALRIs) in children worldwide and a major contributor to hospital admissions among young children, placing a significant burden on healthcare systems [78]. Viral infection can also lead to NP in children [79,80]. Viral infections can lead to NP from coinfection with bacterial infections, specifically Staphylococcal or pneumococcal infections. In an autopsy study of 13 children with ventilator-associated pneumonia, nine patients showed histologic features of viral infection. Human adenovirus and RSV were detected. Some of these autopsies showed features of NP [80].

## 6. Diagnosis

### 6.1. Microbiology

Microbiological diagnosis is critical for guiding targeted antimicrobial therapy. The diagnostic yield and clinical utility of various approaches depend on the timing of specimen collection, prior antibiotic exposure, and availability of advanced molecular techniques.

#### 6.1.1. Specimen Selection and Conventional Methods

The highest yield specimen diagnostically is a direct sampling of pleural fluid, lung aspirate, or bronchoalveolar lavage fluid (BALF) [16,17,25]. Blood and sputum cultures are less sensitive, especially after initiation of antibiotics, but should still be obtained in all hospitalized patients [15,16,23,47]. In a large pediatric cohort, pleural fluid cultures and pneumococcal antigen testing were the most effective for identifying *Streptococcus pneumoniae*, the leading causative agent in pediatric NP [16,25]. However, even with optimal sampling, a conventional culture method only yields a microbiological diagnosis in 30–50% of cases, mainly due to previous antibiotic exposure [16,17,25].

#### 6.1.2. Antigen Detection and PCR

Antigen detection (e.g., pneumococcal capsular antigen in pleural fluid) and targeted PCR assays can increase diagnostic sensitivity, particularly in those with *S.pneumoniae* and fastidious, slow-growing, anaerobic pathogens [25,27,81,82]. In one study, patients with empyema and culture-negative pleural fluid, 31% yielded a positive result with 16S rRNA PCR [81].

#### 6.1.3. Metagenomic Next-Generation Sequencing (mNGS)

mNGS is a powerful tool for comprehensive pathogen detection in pediatric pneumonia, particularly in cases refractory to standard therapy or with negative conventional diagnostics. mNGS can be applied to BALF, pleural fluid, lung tissue, or blood. They can identify a broad spectrum of bacterial, viral, and fungal pathogens with significantly higher sensitivity than traditional methods [83,84,85]. One study demonstrated a positive detection rate of >90% in children with severe or non-responding pneumonia. It is particularly advantageous in those who have previously received antibiotics or when rare or unexpected pathogens are suspected [83,84,85]. However, the interpretation of mNGS requires clinical correlation, as the detection of microbial DNA does not always equate to active infection. Quantitative thresholds, host inflammatory markers, and integration with clinical findings are essential to differentiate colonization from true infection [83,84,85]. Adjustments to antimicrobial regimens based on mNGS and conventional results have been associated with improved clinical response and reduced unnecessary antibiotic exposure [83,85].

### 6.2. Radiology

Imaging plays a central role in the diagnosis, assessment, and management of pediatric NP. Typically, the first line is a chest radiograph followed by either a lung ultrasound (Lung US) or chest computed tomography (Chest CT). All of these can be used to detect necrosis and complications, though they each have different diagnostic yield, safety profiles, and costs associated with them.

**Figure 3:**
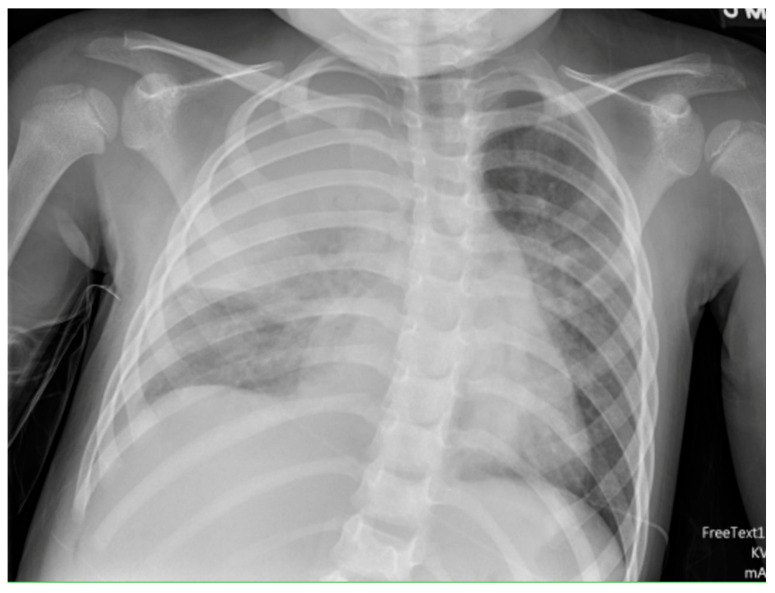
Necrotizing Pneumonia on Chest Radiograph. Plain chest radiograph showing consolidation in the right upper lobe.

**Figure 4:**
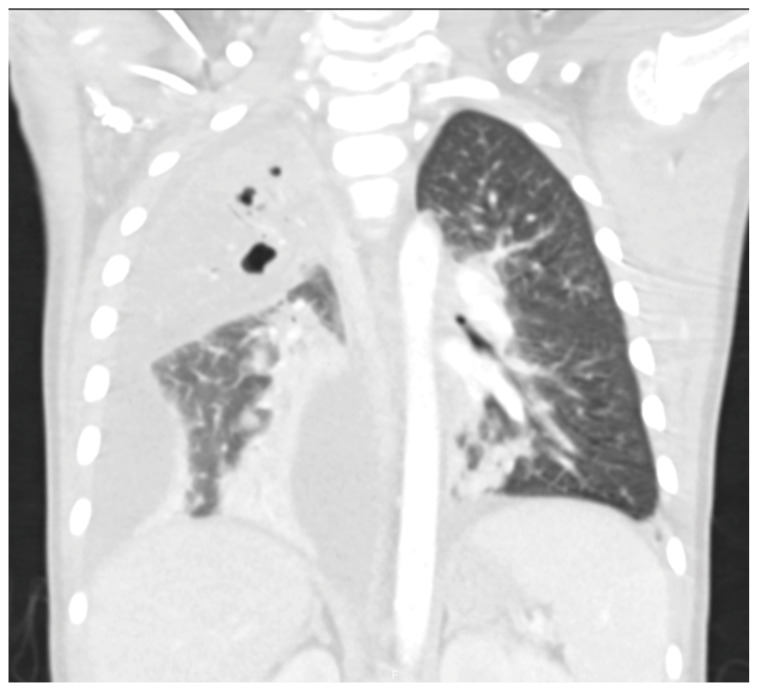
Necrotizing Pneumonia on Chest CT. Right upper lobg necrotizing pneumonia with cavitary changes and pneumotoceles. Moderate to large complex right parapneumonic effusion. Seen with lung windows.

**Figure 5:**
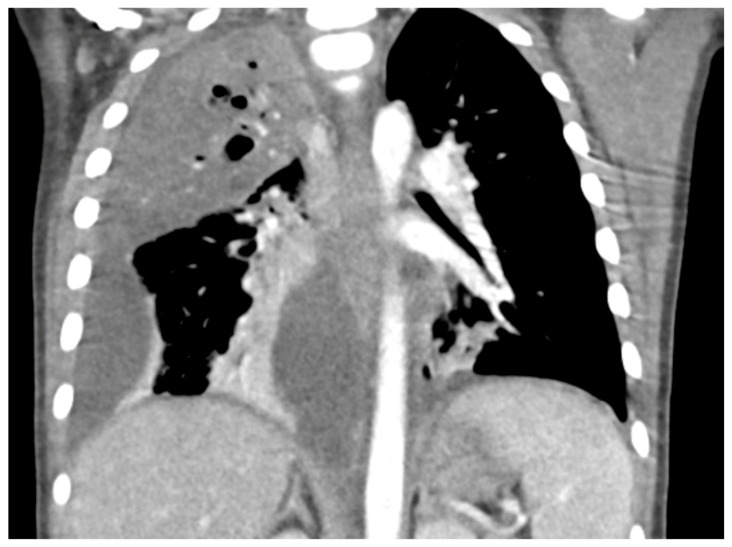
Necrotizing Pneumonia on Chest CT. Right upper lobg necrotizing pneumonia with cavitary changes and pneumotoceles. Moderate to large complex right parapneumonic effusion. Seen with contrast-enhanced windows.

**Figure 6:**
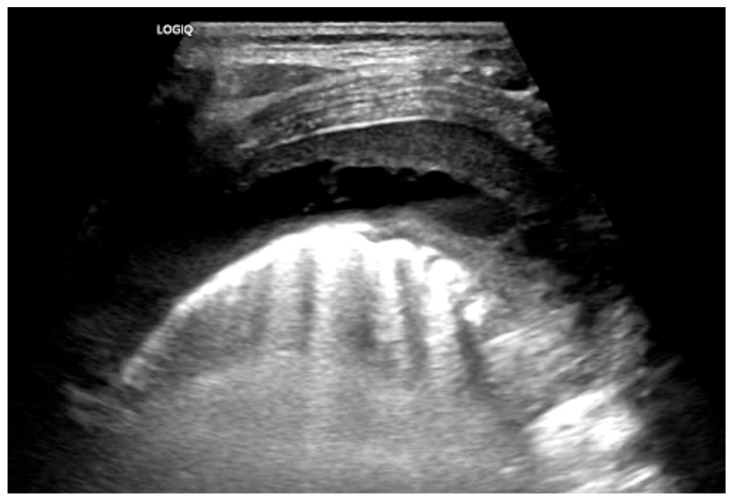
Complex Parapneumonic Effusion on Lung US. Complex parapneumonic effusion with loculations.

Chest radiographs are the first line for patients with suspected complicated or severe pneumonia. They are quick, low-cost, and have low radiation exposure [13,23,86,87]. In NP, they often show a dense consolidation, loss of normal lung architecture, possible air-fluid levels, pneumatoceles, pleural free air (bronchopleural fistula), and pleural effusions. However, it may underestimate the extent of parenchymal destruction or miss early cavitation. It also cannot characterize a pleural effusion beyond size [13,69,86,88,89,90]. Chest CT defines NP better than chest radiograph [91]. Though it is inferior to Lung US or Chest CT, a chest radiograph is a good screening exam that can suggest the need for additional imaging. Chest CT is reserved for atypical clinical courses, complications, or pre-procedural planning. See Figure 3, Figure 4, Figure 5 and Figure 6 for examples of NP on chest radiograph, Chest CT, and complex effusion on Lung US from the same patient.

Lung US has shown similar sensitivity to Chest CT in the diagnosis and identification of necrotizing pneumonia [92,93]. Though both modalities identify pleural effusions with equal accuracy, Lung US is superior at identifying septations (20.4% on Chest CT vs. 62.5% on Lung US) [92,94]. Septations on imaging imply fibrin deposition in the pleural space and have a moderate correlation with purulence. This is important as empyema usually requires a drainage intervention in addition to antibiotics [38]. Lung US also has the benefit of having no radiation exposure and a lower cost [92]. Doppler can be used to identify areas of impaired perfusion and predict large areas of necrosis prior to Chest CT changes [94]. However, Chest CT can detect and differentiate types of parenchymal cavities more easily than Lung US, though it does have a higher cost and significant radiation exposure [93]. Overall, given the lower cost and lack of radiation exposure, Lung US should be the first line to diagnose and manage NP. Hence, Chest CT is not routinely recommended for the management of an NP [38]. Low-dose Chest CT with IV contrast should be used for severe cases that are not following the typical course or to evaluate for other complications, such as lung abscess, bronchopleural fistula, or for pre-surgical planning [92]. Below are four images of the same four-year-old patient with necrotizing pneumonia, imaged via different modalities.

## 7. Management

### 7.1. Antibiotics

Empiric intravenous antibiotics are the cornerstone of necrotizing pneumonia management. Therapy should be initiated promptly and tailored to the most common pathogens, *Streptococcus pneumoniae* and *Staphylococcus aureus* [17,23,95,96]. In those patients with complex chronic conditions, coverage for *Pseudomonas aeruginosa* should be considered, given that it is more likely in that specific population [14]. A multicenter study from the U.S. reported that MRSA coinfection in children with influenza is associated with high mortality among critically ill patients. Early addition of a second anti-MRSA agent to vancomycin improved outcomes, supporting the use of dual anti-staphylococcal therapy in suspected severe cases [97]. The regimen should be adjusted based on local resistance patterns and any microbiological data obtained [17,23,25,96].

Narrowing therapy to a specific organism is often difficult, as the aetiologic agent is frequently not identified [13,24]. In practice, regimens often include a third-generation cephalosporin plus clindamycin or vancomycin if MRSA is suspected [23,26,96]. Though vancomycin is often used, it may not be the preferred anti-MRSA antibiotic given its lack of lung penetration [12,98,99]. There is evidence that treatment with clindamycin or linezolid for community-acquired *Staphylococcus aureus* is associated with improved outcomes; likely due to their protein-inhibiting abilities, as NP is often caused by PVL-producing strains of *S. aureus* [63]. The duration of intravenous therapy is typically prolonged and often followed by an oral course to complete a total of 3–4 weeks, or at least 2 weeks after the patient becomes afebrile [12,23,95,96]. Children with NP require a longer duration of oral antibiotics than those with pneumonia without necrosis [100].

### 7.2. Management of Pleural Involvement

Pleural involvement is frequent in NP, with many children developing moderate to large effusions or empyemas [13,17,23,25]. In a survey, it was found that there is a lack of consensus on the optimal management for PPE [101]. Small to moderate-sized PPE in children may be managed without a chest tube while not increasing complications [102]. Management is guided by the type of effusion, size, and clinical impact of the effusion (Table 5). The American College of Chest Physicians guidelines suggest repeated diagnostic thoracentesis to measure pleural fluid biomarkers to reassess the need for chest tube drainage in adults [103]. This approach is not validated with any outcome data. In children, this is not a typical practice. When indicated, chest tube drainage is the more typical approach in most patients along with appropriate antibiotics.

#### 7.2.1. Tube Thoracostomy

In adults, the American Association for Thoracic Surgery and the Infectious Diseases Society of America recommend a tube thoracostomy (chest tube placement) as initial therapy for moderate to large effusions or empyema, particularly if there is respiratory compromise [7,17,95,96,104]. If the effusion is an empyema, then definitive management should be initiated with a drainage procedure [38]. It is recommended that a chest tube be inserted under image guidance [46,105]. Ultrasound, CT scan, and fluoroscopy are the modalities used for image-guided chest tube insertion. A small-bore chest tube, in general, is sufficient to drain pleural fluid in most patients [46,106].

#### 7.2.2. Fibrinolytics

When the infected pleural space goes into a fibrinopurulent phase, the treatment options include chest tube placement +/− instillation of fibrinolytics vs. a video-assisted thoracoscopic surgery (VATS) [49,107]. Fibrinolysis has been found to be effective and safe as well as superior to chest tube drainage alone, demonstrated in both head-to-head analysis and in patients who failed chest tube drainage alone [38,108,109,110,111]. Fibrinolytic therapy through a chest tube is the preferred mechanism for debridement in these cases, as it does not require an operation and prospective trials have shown that it is equally as effective as operative therapy but is associated with lower hospital costs [38,112,113,114,115].

After instituting hospital guidelines for fibrinolytics (tissue Plasminogen Activator (tPA)) through a small-bore chest tube, the use of VATS has significantly decreased in children with PPE without increasing any failures [116]. However, in a randomized trial from Iran, the VATS procedure showed a favorable performance over intrapleural fibrinolytic administration [117]. In children in the U.S., the use of VATS has decreased without increasing length of stay or need for additional procedures [118].

The recommended initial regimen for fibrinolytics in pediatric empyema patients is three doses of alteplase (tPA) 24 h apart with a one-hour dwell time [38,119]. If there is a lack of clinical improvement either clinically or radiologically, but persistent pleural space disease, clinicians should consider surgical treatment at that point [38,120]. These recommendations; however, are for empyema, in general, and not specific to patients with necrotizing pneumonia. Addition of Dornase Alfa to tPA for intrapleural administration has not been found to be beneficial in a randomized controlled study in children [119].

In a multicenter study, it was found that intrapleural tPA was associated with treatment failure (defined as additional procedure or length of stay longer than 14 days) in a 3rd of patients [121]. In two randomized studies, the failure rate for fibrinolytic therapy was 16% [38,114,115]. In NP, there is a theoretical concern that, given the impaired integrity of the lung parenchyma and pleura, fibrinolytic therapy may lead to bronchopleural fistula. Bronchopleural fistulas are a known complication of NP and are associated with higher mortality and longer length of stay [122,123]. However, recent data demonstrated that fibrinolytic therapy in necrotizing pneumonia is not associated with an increased risk of bronchopleural fistula [26]. Therefore, fibrinolytic therapy should still be used as first-line treatment for NP patients with empyema.

#### 7.2.3. Video-Assisted Thoracoscopic Surgery

Surgical intervention in the form of a VATS procedure is reserved for those who have failed either clinically or due to persistent loculated collections on imaging. In a randomized study from Iran, it was reported that in children with empyema, the VATS procedure was found to have a more favorable outcome compared to chest tube and fibrinolytics [117]. The use of VATS procedure for PPE has decreased as chest drainage with fibrinolytics was found to be as effective as the VATS procedure [113]. VATS is preferred over open thoracotomy due to lower morbidity and shorter recovery times. The need for surgery in NP is uncommon, with most children responding to medical and less invasive interventions.

#### 7.2.4. Decortication

Thoracoscopic debridement is a therapeutic option for managing empyema-associated NP. When used as a primary intervention in children, it is associated with favorable outcomes, providing effective drainage and facilitating full lung re-expansion [124]. Open decortication is rarely required and generally reserved for refractory cases [7,13,17,23,25,95,96,104,125]. The Japanese Association of Thoracic Surgery recommends decortication (both thoracoscopy and open thoracotomy) for acute empyema. However, the recommendation for the intrapleural infusion of fibrinolytic agents for acute empyema is undetermined in their guidelines [126]. Note that fibrinolytics are off-label in Japan [126]. Similarly, the American Association for Thoracic Surgery recommends chest tube placement and a VATS procedure for acute empyema [95]. However, a routine use of fibrinolytics was not recommended [95]. Both these guidelines are for adults and not specific to children.

#### 7.2.5. Surgical Procedures

Surgical indications in the management of NP are not well established. Surgical procedures in the form of decortication, lobectomy, wedge resection, and pneumonectomy are options in selected patients with NP. In a university center in the Czech Republic, out of 1295 children with CAP, 47 patients (3.6%) developed NP, 36 of whom underwent parenchymal lung resection. *Streptococcus pneumoniae* was the most prevalent organism in their series. Long-term follow-up after surgical resection showed normal lung function in 64.8% of cases [127].

Children requiring surgical intervention for NP with massive lung necrosis or cavities involving more than 50% of the affected lobe on computed tomography experienced a more prolonged clinical course and post-operative complications [107]. The authors suggested that lobectomy in such cases may help shorten the post-operative recovery and reduce the need for additional surgeries [107]. Surgical resection with or without decortication is used in immunocompetent as well as immunocompromised children with lung abscesses [128]. A conservative surgical approach of lung necrosectomy, the removal of necrotic lung tissue while preserving adjacent healthy lung tissue, has been advocated by some [129].

### 7.3. Supportive Therapies

There are no consensus guidelines on the management of necrotizing pneumonia or even complicated pneumonia, and children should be referred to a center of expertise [13]. Oxygen therapy is recommended for oxygen saturations below 92% [130]. Additional respiratory support, such as high flow nasal cannula, continuous or bi-level positive airway pressure, and intubation with mechanical ventilation, should be used on a patient-specific basis. However, given the significant risk of pneumothorax and bronchopleural fistula in NP patients, care should be taken to avoid excessive airway pressures in order to reduce the risk of the above complications.

Patients with NP are at increased risk of the syndrome of inappropriate anti-diuretic hormone and therefore at risk of hyponatremia. Though the specific rate of hyponatremia is not known in the pediatric NP population, it has been seen in 33% of children hospitalized with CAP [131]. Given this risk, NP patients should be given isotonic intravenous fluids when they require maintenance fluids, as isotonic fluids are associated with a lower risk of hyponatremia [132].

## 8. Prognosis

Pediatric necrotizing pneumonia is associated with a higher complication rate and severity of illness than typical CAP. Compared with children hospitalized with CAP, children with complicated pneumonia are older, receive higher rates of antibiotic therapy for MRSA and Pseudomonas infections, have a longer hospital length of stay, and have higher rates of ICU admissions and mechanical ventilation [9]. Despite the severity, the long-term prognosis is favorable, especially in previously healthy children [16,48].

Mortality rates are low, generally ranging from 0 to 4% in large series. Deaths are more likely in patients with complex chronic conditions, severe disease (e.g., massive necrosis, septic shock), or in infants under 1 year. In otherwise healthy children, mortality is rare [13,14,15,21,25].

At the 6-month mark, physical exam changes tend to resolve, and exercise capabilities have normalized [15,17,30,48,107]. In terms of imaging, within 6 months, 73% of patients will have complete or near-complete resolution of previously seen lesions. Only a few studies followed patients for more than 6 months, but in those patients, only 5.8% demonstrated residual necrotic areas [15].

Long-term lung function has been less well studied in this patient population. However, these results have been reassuring, with most patients having normalization of lung spirometry. A small percentage of patients did demonstrate mild obstructive or restrictive patterns. However, even those with technically normal spirometry results were on the low end of normal, which could lead to functional impairment as adults [16,48]. In a Finnish study, 26 children with empyema were followed for an average of 8 years (range: 3–19 years). Physical examinations were normal in all participants, and 80% had normal spirometry results. Obstructive airway disease was identified in 16% of the children. Chest radiographs revealed abnormalities in 36% of cases, while lung MRI showed abnormalities in 92% of patients [133].

## 9. Conclusions

Pediatric NP is an uncommon condition but increasingly recognized complication of pneumonia characterized by severe acute illness, prolonged hospitalization, and a high rate of local complications such as pleural effusion, empyema, and bronchopleural fistula [13,16,17,25,47]. The disease course typically necessitates extended antibiotic therapy, and in many cases, pleural drainage procedures with surgical intervention reserved for refractory cases [7,13,25]. Despite these facts, the short and long-term prognosis is generally excellent, with most children achieving full clinical and radiological recovery with minimal long-term sequelae [16,30,48,107].

The microbiological etiology is most commonly *Streptococcus pneumoniae* and *Staphylococcus aureus*, but the spectrum of causative organisms is broadening, and the incidence of NP appears to be rising [14,15]. There is an increase in the prevalence of *Mycoplasma pneumoniae*, especially in East Asian countries. The use of molecular diagnostic techniques, particularly PCR on pleural fluid and respiratory samples, has improved pathogen detection rates and may further refine targeted therapy in the future [13,25]. However, the optimal integration of PCR and other advanced diagnostics into routine clinical practices remains an area for further study.

Limitations of this paper include a lack of randomized controlled trials on the optimal management of this specific sub-population. Most of the management data has to be extrapolated from complicated pneumonia without necrosis.

A key challenge remains the early identification of patients at risk for severe necrosis or those likely to require surgical intervention. Further research to determine which patients will necessitate surgical intervention at an earlier stage could lead to earlier recovery and shorten hospital stay. Additionally, while most children recover fully within 6 months, data on outcomes beyond this period are limited [16,30,48,107]. Longitudinal studies are warranted to assess the potential for late sequelae and the effects of various types of therapies, including subtle pulmonary dysfunction or structural abnormalities.

## Figures and Tables

**Table 1:** Classification of pneumonias * [2,3,4,5,6].

Pneumonia	Definition
Community-acquired pneumonia (CAP)	Pneumonia occurs outside the hospital in patients who were not admitted at least 30 days before the onset. Viral infections account for 80% cases [7,8].
Hospital-acquired pneumonia (HAP)	Pneumonia acquired at least 2 days after hospitalization, not incubating before admission. Predominantly bacterial etiology [8].
Ventilator-associated pneumonia (VAP)	HAP occurring > 48 h after intubation Predominantly bacterial etiology [8].
Healthcare-associated pneumonia (HCAP) (No longer mentioned in 2016 guidelines) [4]	Pneumonia in those who were hospitalized or in a nursing home within 90 days, or home infusion or chronic dialysis within 30 days. Predominantly bacterial etiology [8].

* The frequency of NP in each of the above categories is not reported in the literature.

**Table 2:** Clinical conditions presenting with necrosis in common infectious lung diseases [10,11].

Clinical Entity	Clinical Features	Management
Lung abscess	Solitary abscess, indolent course.	Antibiotics with or without surgical resection (uncommon).
Necrotizing pneumonia	Consolidated lung with necrosis and multiple small abscesses, which may coalesce to form large abscesses, pneumatoceles, bullae, pneumothorax, bronchopleural fistula, and PPE. Usually presents with fever, elevated inflammatory markers, sepsis, and respiratory failure.	Antibiotics, supportive therapy, chest tube, and, rarely, surgical procedures.
Pulmonary gangrene	Progressive devitalization of lung tissue with at least 50% or more of a lobe is necrotic. It can be from the progression of NP.	Antibiotics and surgical procedures (e.g., lobectomy) are more often needed.

**Table 3:** Microorganisms known to cause necrotizing pneumonia [23].

Bacteria	Fungi	Viruses
*Streptococcus pneumoniae* *	*Aspergillus* species	Influenza
*Staphylococcus aureus* *	*Candida* species	Adenovirus
*Streptococcus mitis* species *	*Histoplasma caspulatum*	Herpes viruses, including Cytomegalovirus
*Mycoplasma pneumoniae*	*Coccidoides* species	Varicella-Zoster
*Streptococcus pyogenes*	*Blastomyces* species	Ebstein-Barr Virus
*Pseudomonas* species	*Cryptococcus neoformans*	
*Fusobacterium* species		
*Hemophilus influenzae* [30]		
*Klebsiella* species [31,32]		
*Escherichia coli* [33]		
Brusellosis [34]		

* Most common organisms causing necrotizing pneumonia.

**Table 4:** The Light criteria for exudative effusion [39,40].

Pleural Fluid Examination		Sensitivity (%)	Specificity (%)
Pleural: Serum Protein Ratio	>0.5	86	84
Pleural: Serum Lactate Dehydrogenase Ratio	>0.6	90	82
Pleural Fluid Lactate Dehydrogenase Level	>2/3 Upper Limit of Serum LDH	82	89
Any one of the above criteria		98	83

The Above data is taken from adults, and much of the pediatric evidence is extrapolated from adult data.

**Table 5:** American College of Chest Physicians staging of pleural infection and drainage recommendations.

Stage	Features of Effusion	Thoracentesis/Chest Tube Drainage
Stage I: Uncomplicated PPE	Minimal, free-flowing, <10 mm thickness on lateral decubitus	No/No
Stage II: Uncomplicated PPE	Small to moderate, free-flowing, >10 mm and less than half of the hemithorax	Yes/No
Stage III: Complicated PPE	Large, free-flowing, ≥half of the hemithorax, loculation/septation	Yes/Yes
Stage IV: Empyema	Thick pus	Yes/Yes

PPE: paraoneumonic effusion; Adapted from the reference [46].

## Data Availability

No new data were created or analyzed in this study. Data sharing is not applicable to this article.

## Abbreviations

The following abbreviations are used in this manuscript:

BALF	Bronchoalveolar Lavage Fluid
CAP	Community-acquired Pneumonia
CT	Computer tomography
HAP	Hospital-acquired Pneumonia
HCAP	Healthcare-associated Pneumonia
mNGS	Next-Generation Sequencing
MRSA	Methicillin-Resistant Staphylococcus aureus
MSSA	Methicillin-Sensitive Staphylococcus aureus
NP	Necrotizing Pneumonia
PVL	Panton-Valentine Leucocidine
tPA	Tissue Plasminogen Activator
U.S.	United States
US	Ultrasound
VAP	Ventilator-associated Pneumonia
VATS	Video Assisted Thoracic Surgery
